# Coronally advanced flap and connective tissue graft with or without plasma rich in growth factors (PRGF) in treatment of gingival recession

**DOI:** 10.4317/jced.54573

**Published:** 2018-05-01

**Authors:** Niloofar Jenabian, Mina Motallebnejad, Ehsan Zahedi, Nima D. Sarmast, Nikola Angelov

**Affiliations:** 1 Oral Health Research Center, Babol University of Medical Sciences, Babol, Iran; 2 University of California Los Angeles, School of Dentistry, Los Angeles, USA; 3 Department of Periodontics and Dental Hygiene, The University of Texas School of Dentistry at Houston, Houston, Texas, USA

## Abstract

**Background:**

Several researchers have tried to improve the results of gingival recession treatment techniques. One of the methods is to use growth factors The present study was undertaken to evaluate the effect of CAF (coronally advanced flap) + CTG (connective tissue graft) + PRGF (plasma rich in growth factors) in the treatment of Miller Class I buccal gingival recession.

**Material and Methods:**

Twenty-two teeth with Miller Class I gingival recession in 6 patients 26 ‒ 47 years of age were included in a split-mouth designed randomized controlled trial (RCT). In each patient, one side was treated with CAF + CTG + PRGF (test) and the other side was treated with CAF + CTG (control). The following parameters were measured before surgery and up to 6 months after surgery on the mid-buccal surface of the tooth: keratinized tissue width (KTW), clinical attachment level (CAL), probing depth (PD), vertical recession depth (VRD), recession depth (RD), gingival thickness (GT), root coverage in percentage (RC%) and the distance between the CEJ and mucogingival junction (MGJL). Data were analyzed with paired t-test and repeated measures ANOVA.

**Results:**

After 6 months noticeable improvements were observed in both groups in all the variables measured except for PD; however, the differences between the two groups were not significant. RC% was 80 ± 25% and 67 ± 28% in the test and control groups, respectively, after 6 months.

**Conclusions:**

Both CAF + CTG + PRGF and CAF + CTG treatment modalities resulted in favorable root coverage; however, the addition of PRGF added no measurable significant effect.

** Key words:**Connective tissue graft, dental root coverage, gingival recession, growth factors, mucogingival surgery, periodontal plastic surgery.

## Introduction

Gingival recession has been defined as the migration of marginal soft tissue to a point apical to the tooth or the platform of a dental implant and is prevalent in between 20-100% in the general population (1,2) (Löe *et al.* 1994, Albandar & Kingman 1999). Gingival recession can result in tooth hypersensitivity, pain and difficulty in oral hygiene procedures, root surface caries, unaesthetic appearance of the gingiva and loss of periodontal attachments (3-5) (Rees & Addy 2002, Goutoudi *et al.* 1997, Oliver *et al.* 1998). Interestingly, gingival recession is also a common occurrence in communities with high oral hygiene standards, which is manifested by denuding of the buccal surface of the root (6) (Wennström 1996). The exact mechanism of gingival recession is not fully understood. Some researchers have suggested tooth abrasion (7) (Litonjua *et al.* 2003), improper tooth position, tooth eruption path, form and position of the tooth in the dental arch, dehiscence of the alveolar bone, muscular attachment and frenal pull, periodontal disease and its treatment, inappropriate prosthetic or surgical treatments (iatrogenic), improper oral hygiene techniques (such as brushing, flossing and use of interdental brushes) and other habit-related behaviors such as oral piercing as some of the factors resulting in gingival recession (8) (Wennström 1996). The most important factor increasing the odds of gingival recession is a thin biotype of the gingiva, in which a thin marginal gingival tissue covers the vessel-free surface of the root (9) (Müller *et al.* 1998). Periodontal plastic surgeries aiming to cover the root can be divided into two groups: pedicled soft tissue graft (laterally displaced flap, coronally displaced flap and subepithelial connective tissue graft) and free soft tissue graft (autogenous free gingival tissue graft and autogenous free connective tissue graft). Autogenous free connective tissue can be harvested from the palate or edentulous ridge areas. High durability of subepithelial connective tissue grafts is attributed to the presence of two blood supplies, i.e. the facial gingival flap and the exposed tissue in the denuded area of the root (10) (Langer & Langer 1985). Nelson suggested the use of a full-thickness flap to cover SCTG (11) (Nelson 1987). However other studies showed no difference from those of a partial thickness flap (12) (Mazzocco *et al.*, 2011). Raetzke (13) suggested the envelope technique in which SCTG is placed in the space between the partial thickness flap and the denuded surface of the root, with or without a strap of marginal epithelium, without vertical releasing incisions (Raetzke 1985). Placement of the connective tissue between the flap and the denuded surface of the root is referred to as the “bilaminar technique” (14) (Cordioli *et al.* 2001). Coronally advanced flap (CAF) in conjunction with the connective tissue graft (CTG) is considered the gold standard of treatment of gingival recession due to its high predictability of the treatment results (8,15) (Wennström & Zucchelli 1996, Paolantonio 2002). Anitua introduced a new technique to prepare plasma rich in growth factors (PRGF) (16). This preparation technique is 100% autologous, resulting in plasma rich in biologic mediators to accelerate reconstruction of hard and soft tissues. RPGF, contrary to platelet-rich plasma (PRP), does not contain leukocytes and other inflammatory by-products (11) (Nelson 1987). Its activation with sodium chloride leads to the formation of a polymerized fibrin matrix and release of a number of growth factors. Adhesive molecules derived from plasma, such as fibrinogen, vitronectin and thrombospondin-1, function as a matrix or a scaffold and attract precursor cells and platelets. Platelets are a rich source of growth factors such as PDGF, TGF-β, VEGF, FGF, IGF and GM-CSF (17) (Anitua *et al.* 2010). An *in vitro* study showed that PRGF induces a proliferative response in fibroblasts (18) (Anitua *et al.* 2009). In addition, its effects have been substantiated in improving healing of the epithelial tissue (19) (Anitua 2001), muscle and tendon17 (Anitua *et al.* 2010). The effect of PRGF has also been shown in promoting proliferation, migration and chemotaxis of human osteoblasts (20) (Anitua *et al.* 2013). Recently, PRGF has been demonstrated to have a strong stimulatory effect on human gingival fibroblast (HGF) cell viability and proliferation when compared to platelet rich fibrin (PRF) (21) (Vahabi *et al.* 2015). Given the advantages of PRGF over other techniques of isolating growth factors, its adjunctive use in the treatment of gingival recession can be a possible method of choice when additional biological factors are warranted. The aim of the present study was to evaluate the effect of PRGF on the results of root coverage procedures with the use of CAF+CTG.

## Material and Methods

**Figure 1:**
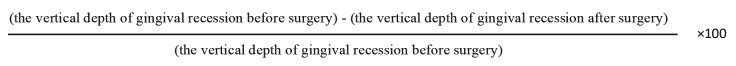
Formula.

A total of 22 teeth with Miller Class I gingival recession, in 6 patients 26‒47 years of age were treated at the Babol University of Medical Sciences. All the treated sites were in the mandible, involving 4 incisors, 2 canines, 8 first premolars and 10 second premolars. A randomization table was used to assign each surgical site in each subject to one of the two treatment groups by flipping a coin. One side was designated as the control side and was treated with connective tissue graft in conjunction with coronally advanced flap (CAF+CTG), and the other side in the same patient was designated as the case side and treated plasma rich in growth factors (PRGF) in addition to the connective tissue graft and coronally advanced flap (CAF+CTG+PRGF). The randomized surgical technique decided was placed in an envelope and submitted to the surgeon immediately before surgery. All the surgical procedures were carried out by one surgeon. The inclusion criteria were: age ≥18 years, acceptable oral hygiene (O’Leary plaque score ≤20%), presence of facial bilateral solitary Miller Class I gingival recessions of ≥2 mm measured from the CEJ on vital anterior or premolar teeth, no dental restorations, absence of bleeding on probing (BOP), keratinized gingival width of ≥2 mm and gingival thickness of ≥0.5 mm (measured at a distance of 2 mm from the apical gingival margin). The exclusion criteria were: pregnancy, coagulation and hematologic disorders, use of antibiotics during previous 6 months, known allergy to materials used during the surgical procedure, active infectious diseases, use of medications interfering with wound healing processes (corticosteroids, antineoplastic agents) or interfering with the function of platelets (NSAIDs), smoking, traumatic tooth brushing habits, use of hard toothbrushes or abrasive toothpastes, frenal pull at the surgical site, a history of periodontal surgery in the area involved during the previous 2 years, use of a removable prosthetic appliance in the area involved and use of medications causing gingival hyperplasia. The protocol of the study was approved by the Ethics Committee of Babol University of Medical Sciences. The clinical parameters were measured using a standard Williams probe (HU-Friedy, Chicago, IL, USA) by a periodontist blinded to the treatment modality in the area involved. The clinical parameters measured were: KTW (the distance between the free gingival margin and mucogingival junction), CAL (the distance between the cemento-enamel junction (CEJ) and the gingival sulcus floor at the mid-buccal surface of the tooth), PPD (the distance between the free gingival margin and the gingival sulcus floor at the mid-buccal surface of the tooth), VRD (the distance between the CEJ and the free gingival margin [the midpoint of the denuded gingival surface] at the mid-buccal surface of the tooth), RW (the width of recession at 1 mm apical to the CEJ in the mesiodistal dimension), GT (the thickness of the gingiva at 2 mm apical to the gingival margin on the buccal aspect determined by penetrating a #15 endodontic file with a silicone disk), MGJL (the distance between the CEJ and MGJ on the mid-buccal area of the tooth involved), The root coverage percentage RC% was determined using the formula below: (Fig. 1).

The wound healing index (HI) was also calculated based on the standard Landry criteria (22) (Landry *et al.* 1988). In short, Healing Index 1 (Very Poor): has 2 or more of the following: tissue color: >= 50% of gingiva red, response to palpation: bleeding, granulation tissue: present, incision margin: not epithelialized, with loss of epithelium beyond incision margin, or suppuration present. Healing Index 2 (Poor): tissue color: >= 50% of gingiva red, response to palpation: bleeding, granulation tissue: present, and incision margin: not epithelialized, with connective tissue exposed. Healing Index 3 (Good): tissue color: >= 25% and < 50% of gingiva red, response to palpation: no bleeding, granulation tissue: none, and incision margin with no connective tissue exposed. Healing Index 4 - Very Good: tissue color: < 25% of gingiva red, response to palpation: no bleeding, granulation tissue: none, and incision margin with no connective tissue exposed. Healing Index 5 (Excellent): tissue color: all tissues pink, response to palpation: no bleeding, granulation tissue: none, and incision margin with no connective tissue exposed (22) (Landry *et al.* 1988).

The pain (PVAS) and esthetic (EVAS) indexes were determined based on visual analog scale (VAS)23 (McCormack *et al.* 1988). In short, the patient was instructed to select a number from 1 to 10, with “10” indicating the greatest pain intensity and the most esthetic appearance of the gingiva and with “1” indicating absence of pain, and the most un-esthetic appearance of the gingiva in patient’s opinion, respectively. KTW, MGJL, PPD, CAL, GT and EVAS were determined before surgery and at 6-week and 6-mointh post-operative intervals. VRD and RW were evaluated before surgery and at 2-week and 6-month post-operative intervals. PVAS was evaluated at 1-, 3- and 7-day post-operative intervals. HI was evaluated at 1-, 3-, 7- and 30-day post-operative intervals. RC% was determined 6 months after surgery. Preparation of PRGF was carried out using the technique described by Anitua & Andia in 2001 (19). Before surgery, 20 mL of the patient’s venous blood was taken and placed in 5 mL test tubes containing 3.8% sodium citrate as an anticoagulant. The tubes were centrifuged for 8 minutes at room temperature (PRGF-Endoret System IV Biotechnology Institute, Vitoria, Spain). After centrifugation, the contents of each tube were divided into the following parts.

1. Plasma with a small amount of growth factors in the uppermost part of the test tube, with a volume of 1 mL (PRGFs).

2. Plasma containing some growth factors with a volume of 0.5 mL (PGFs).

3. Plasma rich in growth factors between the second segment and the white blood cell layer, with an approximate volume of 0.5 mL (PRGF).

4. A white layer of WBCs between the PRGF segment and red blood cells, with a volume of 50 µL.

5. RBC segment.

The first and second segments were removed with a 500-µL pipette and placed in separate test tubes. To achieve great accuracy and prevent any mixing of the PRGF and WBC layers, the third layer was removed in 5 rounds and placed in another test tube using a 100-µL pipette. Then the activation procedure was carried out by adding 50 µL of 10% calcium chloride (PRGF-Activator, Biotechnology Institute) to each mL of PRGF.

Surgical technique: After administration of local anesthesia with 2% lidocaine containing epinephrine (1:80,000) via infiltration technique, sulcular incisions were made with a #15c scalpel blade, which ended at the mesial and distal areas, in two horizontal incisions approximately 2 mm below the papilla followed by mesial and distal vertical releasing incisions which extended beyond the MGJ. The width of the flap in the mesio-distal dimension was wider than the width of the lesion up to half the tooth size. The flap was then elevated in the corono-apical direction with a split-full-split design. First, the surgical papilla was elevated in split design and this separation was extended up to the hypothetical line connecting the probing depth of the two adjacent teeth. The gingival tissue apical to the root exposure area was elevated in a full-thickness fashion in order to provide adequate thickness for graft coverage. The remaining parts of the interdental papilla were de-epithelialized in order to provide an appropriate bed of connective tissue for suturing of the papilla. In the test group, the connective tissue harvested from the palate on the surgery side (incisions were made based on Type A Class II using the Liu & Weisgold classification) (24) (Liu & Weisgold 2002) was immediately coated with PRGF and placed on the root surface (CAF+CTG+PRGF). In the control group, no PRGF was used and only CAF with the use of CTG was performed. The thickness of the connective tissue grafted in all the cases was 1.5 mm, as measured by tissue calipers. In all cases, the connective tissue was stabilized on the denuded root surface with resorbable 5-0 sutures (5.0 Vicryl, Ethicon, Johnson & Johnson, Somerville, NJ) using the sling technique. Subsequently, the flaps were coronally advanced and secured with resorbable 4-0 sutures, using the sling technique, to allow for complete coverage of the graft. Finally, non-eugenol periodontal pack was used for dressing in all cases (Coe-Pack, GC America. Inc., Alsip. IL, USA). All the patients were instructed to use ice-pack immediately after surgery in 20-minute intervals up to 24 hours. The patients were instructed to use 0.12% chlorhexidine mouthwash (Emad Pharmaceuticals, Isfahan, Iran) twice daily for 4 weeks and not brush the surgical site for two weeks. Ibuprofen tablets, 400 mg (Hakim Pharmaceuticals, Tehran, Iran), were prescribed; t.i.d. for 7 days. The sutures were removed after 14 days and plaque control continued with chlorhexidine mouthwash for another two weeks; then the patients were asked to gently resume brushing the area with a soft toothbrush, using the roll technique. During the follow-up sessions, supragingival plaque was removed and oral hygiene instructions were repeated. The patients were recalled at the pre-determined time intervals for the measurement of clinical parameters.

Statistical analysis: All statistical analyses were carried out using SPSS 20 (SPSS Inc., Chicago, IL, USA). Changes in the clinical parameters and variables were evaluated at the time intervals mentioned previously. The means and standard deviations of all the clinical variables in each treatment group were calculated. Kolmogorov-Smirnov test was used to confirm normal distribution of data. Paired samples t-test was used to evaluate difference between the groups and within each group before and after treatment. Repeated measures ANOVA was used to evaluate changes in different parameters. Statistical significance was defined at *P*<0.05.

## Results

All the patients completed the surgical phase of the treatment and the follow-up sessions for 6 months. No cases of flap necrosis, infection or unusual hemorrhage were observed in the patients.

**Figure 2:**
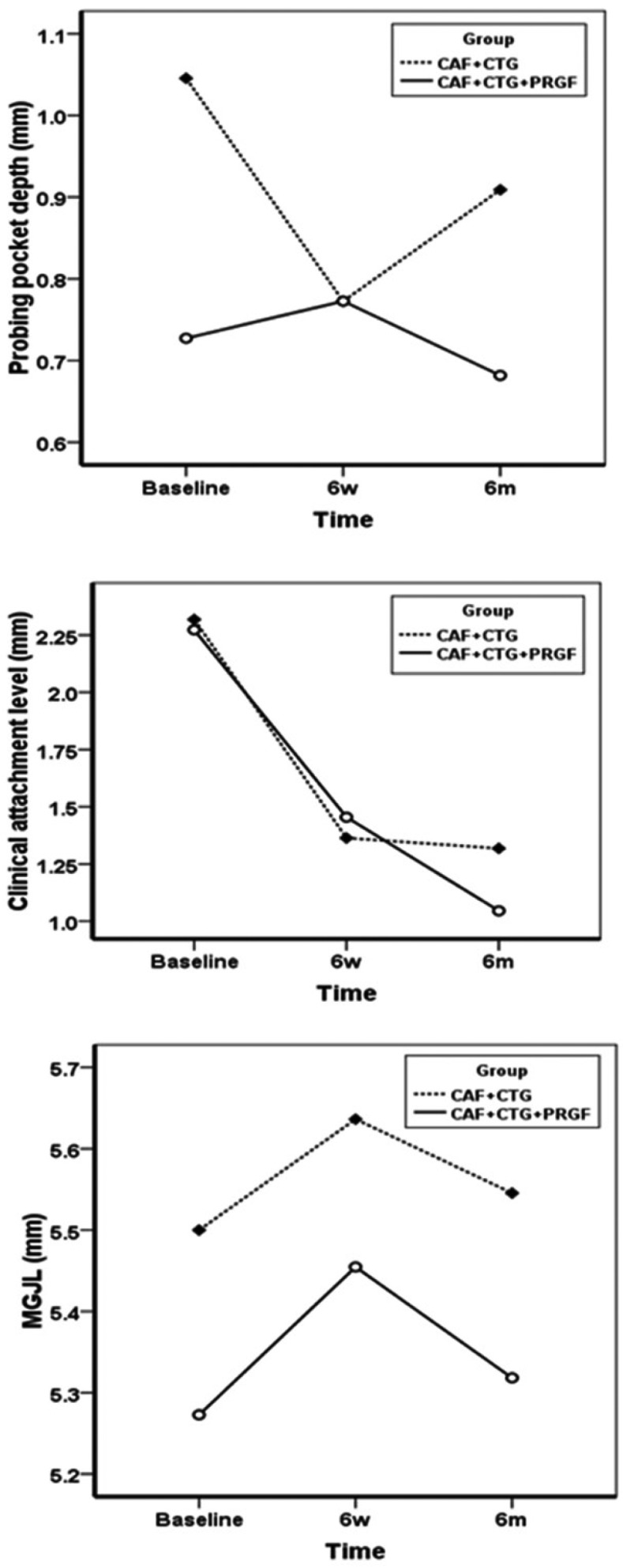
PPD, CAL, MGJL at baseline, 6 weeks and 6 months.

**Figure 3:**
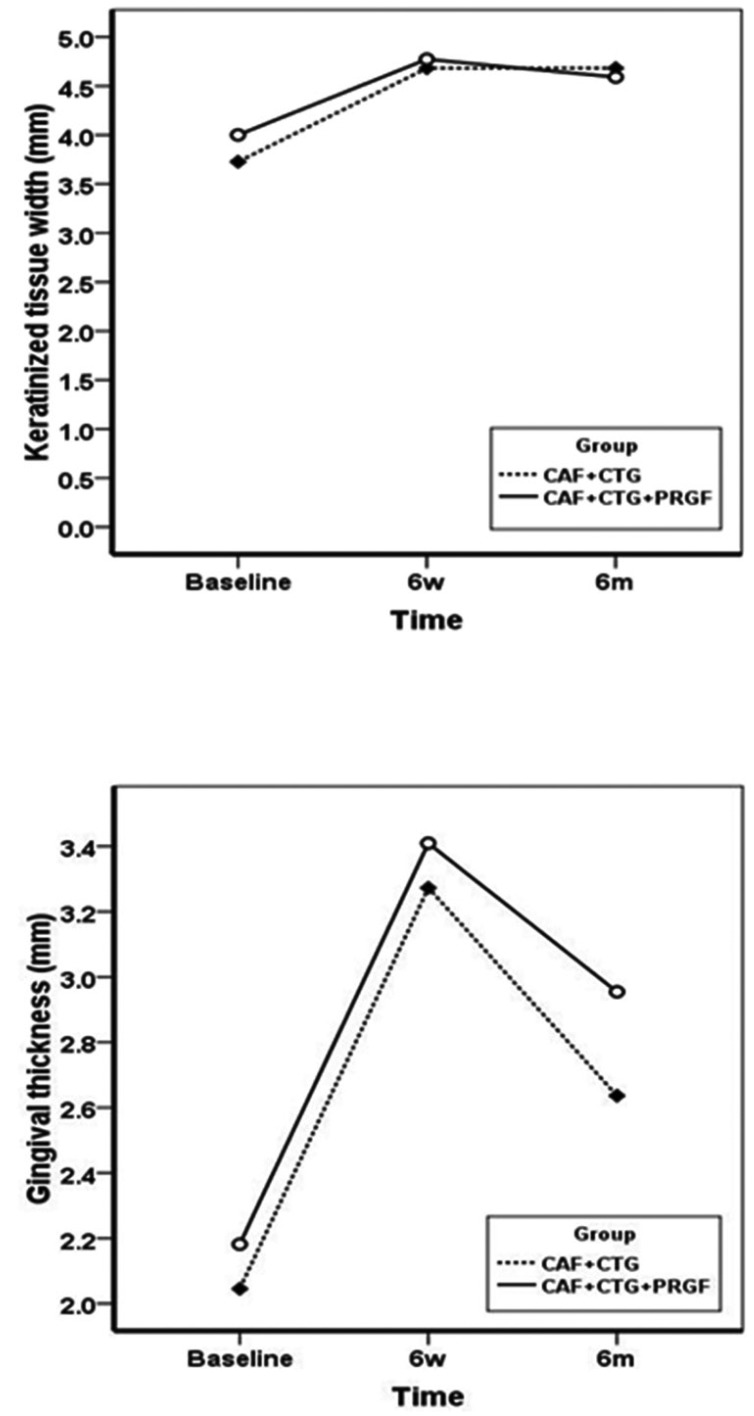
KTW and GT at baseline, 6 weeks and 6 months.

**Figure 4:**
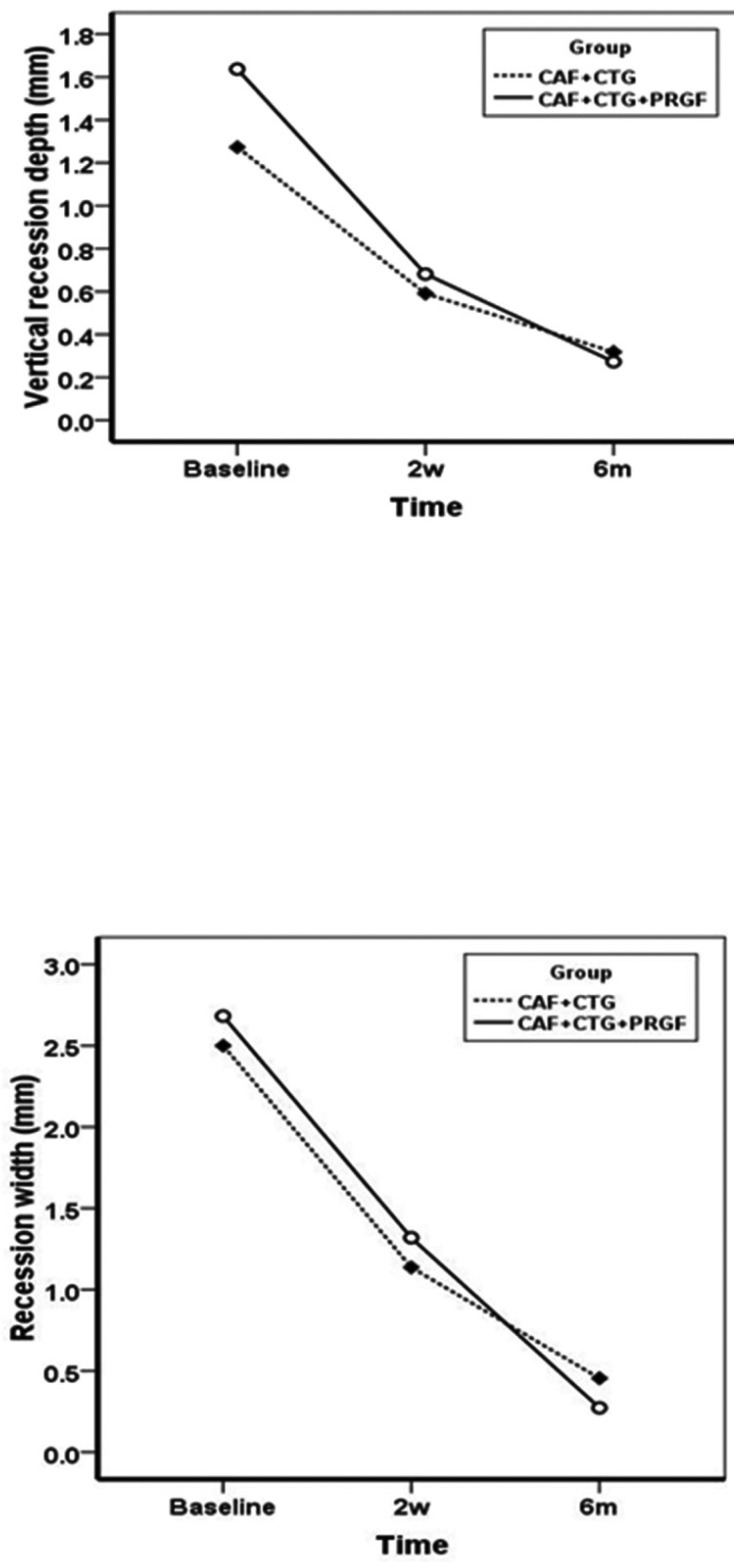
VRD and RW at baseline, 6 weeks and 6 months.

**Figure 5:**
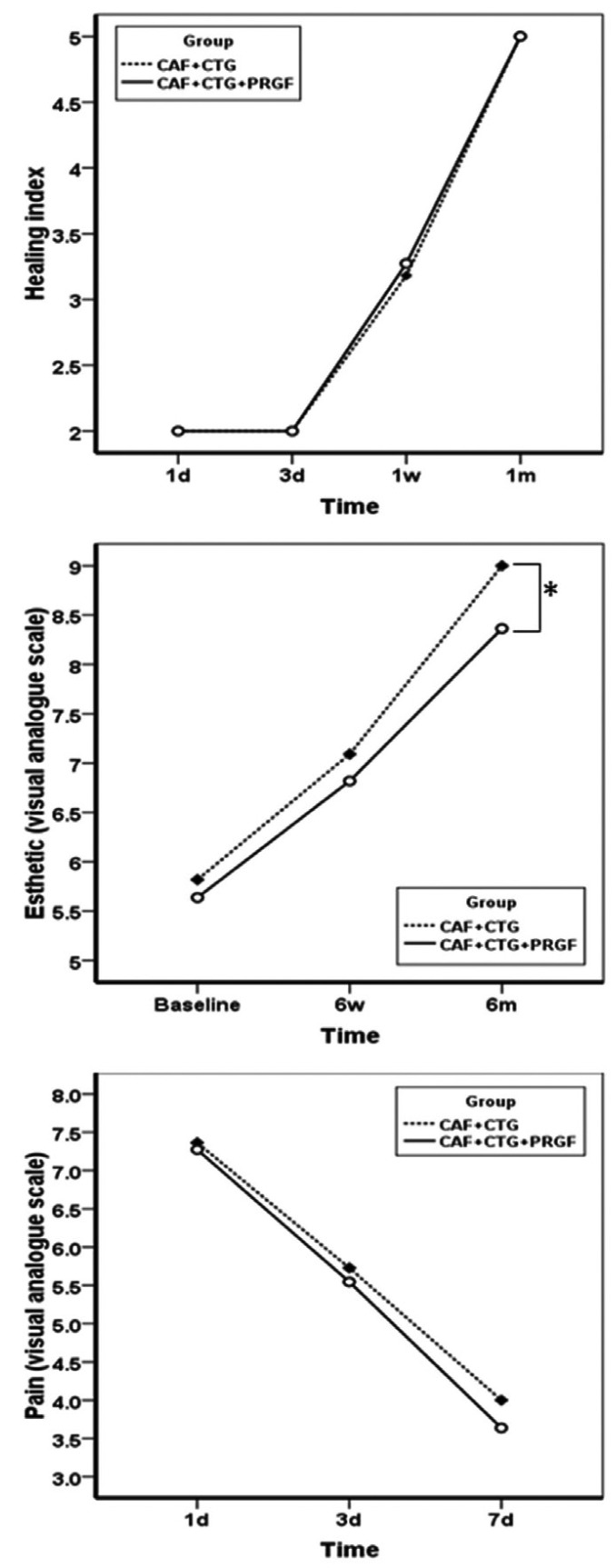
HI from day 1 up to 1 month post-operatively. EVAS at baseline, 6 weeks and 6 months. PVAS at day 1, 3 and 7.

RC% exhibited significant improvements in both groups, with means of 80±25% and 67±28% at 6-month post-operative interval in the test and control groups, respectively; however, after 6 months there were no significant differences between the two groups. The changes in CAL over time within the groups were significant, as expected, but there were no significant changes between the treatment group and the control (Fig. 2). Unlike other variables, PPD did not undergo any significant changes at 6-week and 6-month post-operative intervals (*P*=0.501) within the groups (Fig. 2). Similarly, MGJL, significantly increased in both groups although no statistically significant differences were observed between the experimental and control groups (Fig. 2). The KTW and GT significantly increased in both groups at 6-week and 6-month post-operative intervals, with no statistically significant differences between the two groups (Fig. 3). VRD and RW significantly decreased after 2 weeks and 6 months, with no statistically significant differences between the two groups (Fig. 4). HI exhibited significant differences at 1-, 3-, 7- and 30-day post-operative intervals (*P*<0.0001) with no statistically significant differences between the two groups (Fig. 5). Differences in EVAS and PVAS were significant in each group (*P*<0.0001), but the differences between the two groups, at different time points were not significant (Fig. 5). The only exception was the evaluation of EVAS at 6-month post-operative interval, with significant differences between the two groups (*P*=0.033; Fig. 5).

## Discussion

Several researchers have tried to improve the results of gingival recession treatment techniques. One of the methods is to use growth factors. The majority of studies on the subject have evaluated the effect of PRP on the results of root coverage techniques (25-29) (Shepherd *et al.* 2009, Keceli *et al.* 2008, Petrungaro 2001, Huang *et al.* 2005, Naik *et al.* 2013). However, a new technique has been introduced to extract growth factors without the use of bovine thromboplastin, which results in the preparation of plasma rich in growth factors (PRGF) (30) (Anitua & Andia 2004). In our present study significant improvements were observed in all variables (except for PPD) with no significant differences between the two groups. The only exception was EVAS, which exhibited statistically significant differences between the two groups at 6-month post-operative interval with better results in the control group based on patients’ opinions. Evaluation of a decrease in VRD in a study by Huang *et al.* in 2005 (28) and the effect of PRP+CAF on the treatment of gingival recession lead us to believe that better results have been achieved in the present study, which might be attributed to the use of CTG, differences in growth factors in PRP and PRGF and a higher mean of KTW before surgery. The means of decreases in VRD in the test and control groups in a study by Lafzi *et al.* (2012) (31) were 2.6 mm and 2.3 mm respectively, which are higher than those in the present study. One of the factors which might have contributed to this is the difference in the width of keratinized gingiva before surgery. In the study carried out by Lafzi *et al.*, (31) the mean of this variable before surgery was 4.8 mm before surgery but it was 4 mm in our present study. Pini Prato *et al.* (32) reported that an increase in flap tension results in a decrease in root coverage (Pini Prato *et al.* 2000). In our study the initial means of KTW in the test and control groups were 4 mm and 3.72 mm respectively, highlighting the importance of this factor. The mean percentages of root coverage after surgery were not significantly different between the two groups, consistent with the results of some other studies (26,30,32) Keceli *et al.* 2008, Anitua & Andia 2004, Pini Prato *et al.* 2000). In a study by Huang *et al.* (2005) (28) the mean root coverage percentage in the CAF+PRP group was 81%, which is similar to that achieved in our present study. Miller Class I lesions generally have favorable and predictable conditions for treatment. Several studies have shown favorable results with the use of CTG in the treatment of gingival recession (15,32,33) (Paolantonio 2002, Pino Prato *et al.* 2000, Jankovic *et al.* 2007). Minor differences in the results might be attributed to differences in case selection and the follow-up intervals. The results of the present study, like other studies in which CTG has been used, indicate an increase in the width of keratinized gingiva (34-36) (Zucchelli *et al.* 1998, Caffesse *et al.* 2000, Cheung & Griffin 2004). Based on the results of a study by da Silva (37), when CAF+CTG is used, KTW and GT variables are increased to a greater degree compared to the situation in which only CAF is used (da Silva 2004). On the other hand, Jankovic *et al.* (2007) (33) reported that use of CTG+PRP is more effective than CTG in increasing the width of keratinized gingiva. PRP growth factors might have a positive effect on the proliferation of gingival and periodontal fibroblasts. In addition, PRGF accelerates the reconstruction of gingival connective tissue by stimulating several important processes involved in wound healing (Anitua 2012) (38). It should be kept in mind that CAF alone has a positive effect on KTW because the biologic activity of the granulation tissue is derived from the periodontal ligament (39) (Lundberg & Wennström 1988). In the present study, the mean increase in GT and KTW did not exhibit any significant differences between the test and control groups; though both techniques showed a positive effect on increasing the thickness of gingiva and the width of keratinized gingiva, consistent with the results of other studies (31,37) (Lafzi 2012, da Silva *et al.* 2004). A comprehensive review study evaluated the effect of autogenous platelet concentrates on the clinical results of the treatment of periodontal diseases, reporting no positive effects of these blood products in the treatment of gingival recession (40) Del Fabbro *et al.* 2011). Also in this study, the means of clinical attachment gain in the control (CAF+CTG) and case (CAF+CTG+PRGF) groups were less than those in similar studies (27,31,33) (Petrungaro 2001, Lafzi *et al.* 2012, Jankovic *et al.* 2007), which might be attributed to differences in the thickness of the covering tissue and the conditions of the gingival issue in the mandible, with greater tissue tension and root prominences because in the present study, all the samples were in the mandible and were of the Miller Class I type, which normally gain less attachment level after treatment compared to cases with more advanced gingival recession.

Changes in PPD in both groups the present study were not significant, i.e. PPD did not change during the study period. These conditions are consistent with the results of the majority of studies (Petrungaro 2001, Lafzi *et al.* 2012, Jankovic *et al.* 2007), (27,31,33) but not all of them (22) (Landry *et al.* 1988). Differences in PPD values might be attributed to difference in the techniques used and also difference in the baseline values of PPD in different studies, making it difficult to compare the results. Comparison of EVAS up to the 6-week post-operative interval with the pre-operative situation did not exhibit any significant differences; however, at 6-month post-operative interval there were significant differences between the two groups, indicating the superiority of the control group (CAF+CTG), contrary to the results reported by Cheung *et al.* (2004) (36), demonstrating better esthetic results with the use of platelet concentrates, which might be attributed to the use of a more accurate technique for the evaluation of esthetic results in that study. In that study, three experienced periodontists evaluated the photographs of treatment results and the color, consistency and contour of the gingiva. In our study, evaluation of esthetic results was based on patient judgment using VAS. Evaluation of pain by the patients using VAS did not reveal any significant differences between the two groups. However, in a study by Jankovic *et al.* (33) all the patients treated with CTG reported a high rate of discomfort and during the first 5 days there were significant differences between the two groups in relation to pain severity, with lower pain severity in the CTG+PRP groups (33) (Jankovic *et al.* 2007). Finally, the evaluation of tissue healing with Landry index (Anitua *et al.* 2013) (20) did not reveal significant differences between the two groups, contrary to the results reported by Jankovic *et al.* (33), who reported better initial healing in the PRP group compared to the CTG+PRP group (Jankovic *et al.* 2007) (33). Future randomized controlled studies should include longer follow-up periods and larger sample size to further evaluate the effect of biologics in mucogingival surgery.

## Conclusions

Within its limits, this study showed that treating gingival recession lesions with CAF+CTG versus CAF+CTG+PRGF yields similarly favorable results. The use of PRGF, in addition to CAF+CTG did not yield any additional significant benefits considering healing, major clinical periodontal and soft tissue parameters. Future studies should consider longer follow-up periods and larger sample sizes in conjunction with histological evaluations in order to evaluate the effect of PRGF on reconstruction of periodontal attachments.
